# Unpredicted ecosystem response to compound human impacts in a European river

**DOI:** 10.1038/s41598-024-66943-9

**Published:** 2024-07-16

**Authors:** Jan Köhler, Elisabeth Varga, Stephanie Spahr, Jörn Gessner, Kerstin Stelzer, Gunnar Brandt, Miguel D. Mahecha, Guido Kraemer, Martin Pusch, Christian Wolter, Michael T. Monaghan, Matthias Stöck, Tobias Goldhammer

**Affiliations:** 1 Leibniz Institute of Freshwater Ecology and Inland Fisheries (IGB), Berlin, Germany; 2 Department of Food Chemistry and Toxicology, University of Vienna, Vienna, Austria; 3 Brockmann Consult GmbH, Hamburg, Germany; 4 Institute for Earth System Science and Remote Sensing, Leipzig University, Leipzig, Germany; 5 Remote Sensing Centre for Earth System Research, Leipzig University and Helmholtz Centre for Environmental Research, UFZ, Leipzig, Germany; 6 German Centre for Integrative Biodiversity Research, iDiv, Halle, Jena and Leipzig, Germany; 7 Institute of Biology, Free University Berlin, Berlin, Germany; 8 Present Address: Unit Food Hygiene and Technology, Centre for Food Science and Veterinary Public Health, Clinical Department for Farm Animals and Food System Science, University of Veterinary Medicine Vienna, Vienna, Austria

**Keywords:** Harmful algal bloom, *Prymnesium parvum*, Fish kill, Salinization, River ecology, Multiple stressors, Ecology, Environmental sciences

## Abstract

Climate change elevates the threat of compound heat and drought events, with their ecological and socioeconomic impacts exacerbated by human ecosystem alterations such as eutrophication, salinization, and river engineering. Here, we study how multiple stressors produced an environmental disaster in a large European river, the Oder River, where a toxic bloom of the brackish-water planktonic haptophyte *Prymnesium parvum* (the “golden algae”) killed approximately 1000 metric tons of fish and most mussels and snails. We uncovered the complexity of this event using hydroclimatic data, remote sensing, cell counts, hydrochemical and toxin analyses, and genetics. After incubation in impounded upstream channels with drastically elevated concentrations of salts and nutrients, only a critical combination of chronic salt and nutrient pollution, acute high water temperatures, and low river discharge during a heatwave enabled the riverine mass proliferation of B-type *P. parvum* along a 500 km river section. The dramatic losses of large filter feeders and the spreading of vegetative cells and resting stages make the system more susceptible to new harmful algal blooms. Our findings show that global warming, water use intensification, and chronic ecosystem pollution could increase likelihood and severity of such compound ecoclimatic events, necessitating consideration in future impact models.

## Introduction

Compound events are extreme weather and climate events caused by the combination of interacting physical hazards that impact ecosystems and human well-being^1–3^. These impacts may be amplified by chemical and biological hazards that contribute to cascading effects, and ecosystems chronically pre-stressed by human intervention and management are particularly vulnerable to such compound ecoclimatic events^4^. In rivers, chronic and acute stressors include impoundment, fragmentation, regulation, and pollution. These can interact and lead to catastrophic consequences for socio-ecological systems. Examples include harmful algal blooms (HABs) that endanger freshwater ecosystems by toxin production, elevated pH, or fluctuating oxygen concentrations^5^. Likewise, fish kills are expected to increase with rising air and water temperatures^6^. As their underlying mechanisms are often complex, compound ecoclimatic events have been rarely considered in comprehensive assessments of climate change-associated risks.

HABs significantly affect the structure and functioning of aquatic ecosystems by killing fish at different trophic levels and damaging populations of other benthic and planktonic filter feeders^7^. Fisheries and aquaculture are harmed^8,9^, and human recreational activities and water supplies are severely impacted^10,11^. HABs rely on a multitude of interacting factors: supply of nutrients, light intensities, temperature regimes, losses by grazing, parasitism, sedimentation, and dilution^12^.

Free-flowing rivers are typically characterized by high dilution rates and can only harbor dense blooms of planktonic algae under low flow and slow downstream transport^13^. Reduced summer precipitation and an increasing frequency of record high summer temperatures^14^ result in higher atmospheric water demand and evaporation^1^, leading to drought conditions that naturally imply low river discharge rates with shallower water level and increased light supply. Reservoirs or impoundments further extend the time available for phytoplankton growth and are often essential for the development of stable plankton populations in river systems^15^. Short residence times in rivers, reservoirs or flushed lakes prevent mass-developments under suboptimal conditions, e.g., low temperatures^16^.

The combination of intense land use, industrial pollution, wastewater loading, and low discharge results in ongoing salinization of freshwater ecosystems^17^. Higher salinity is a critical selective factor for salt tolerant algae with reduced species diversity not only at very high and very low salt concentrations, but especially in moderate but variable salinity (brackish water)^18^. HABs usually consist of one or very few toxic species that have outgrown their competitors. Extreme environmental conditions can be a selective advantage for specialists that thrive under reduced competition and pressure from predation, pathogens, or parasites. Low flow, replete nutrients, and high temperatures generally increase the likelihood of HABs^19^. However, accurate predictions of the combined climate-environment interactions triggering HABs remain difficult because of limited knowledge of the physiological demands and ecological interactions of potentially harmful algal species.

Here, we analyze how multiple compounding pressures of climate and human modification led to an unprecedented environmental disaster on the Oder River, an 866 km long lowland river with a catchment of 118,890 km^2^ in the Czech Republic, Poland, and Germany (see Supplementary Figure S1 for a map). This river is highly eutrophic and has been affected by salt emissions from coal and ore mining in the upper catchment (Upper Silesia, upstream of Opole^20^). We synthesized multidisciplinary data and knowledge on the causes and consequences of the bloom of the toxic haptophyte *Prymnesium parvum* that culminated in a massive kill of fish and mollusks in the Oder River in summer 2022. Preliminary data on this fish kill were compiled in government reports^21–23^. Here, we reconstructed for the first time the spatial and temporal development of the algal bloom using data from remote sensing, continuous monitoring stations, and frequent sampling during and after the event. We identified the algal species and genotype, the phytoplankton composition, toxin concentration and structure, and estimated the impacts on fish and mollusk communities. We further discuss the extension of the concept of compound climatic events in light of chronic and acute human-induced stress on river ecosystems resulting in unforeseen biological hazards.

## Results

### Climatic and hydrologic conditions

**Figure 1:**
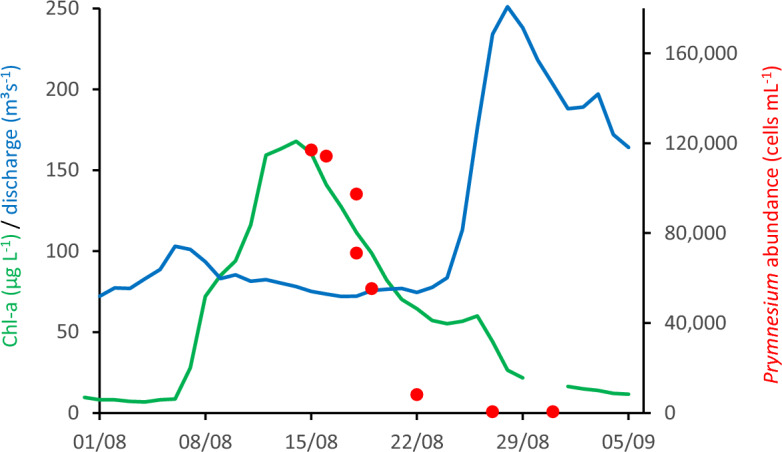
Phytoplankton "peak" development at Frankfurt/Oder (km 584). Green line, Chl-*a* measured fluorometrically, red dots, abundance of *Prymnesium parvum* in counted cells per mL, and blue line, discharge at Eisenhüttenstadt (km 553) in August 2022. Chl-*a* and discharge data provided by the Brandenburg State Office for the Environment^26^. Chl-*a* data re-calibrated by HPLC.

In summer 2022, a drought period that started building up in spring and higher-than-average temperatures (reference period 1991–2020) led to a pronounced drought and heat wave in Central Europe in July^24,25^. In August 2022, the daily means of air temperatures measured at the automated monitoring station in Frankfurt/Oder (river km 584) averaged at 21.9 (14.5–29.5) °C, water temperature at 23.7 (21.6–25.9) °C. Daily average global radiation in this period was 29 to 179 W m^−2^ (mean 128 W m^−2^, see Table 1). The cumulative discharge in the Oder River measured at the gauging station Eisenhüttenstadt (river km 554, measured from the source) from June to August was the lowest on record (1962–2022, Supplementary Fig. S2). Daily means of discharge dropped below 120 m^3^ s^−1^ from June 23 till August 25. In this period, the discharge was always close to the minima and below 50% of the averages observed for these days in 2000–2021 (Supplementary Fig. S3). At the end of August, discharge increased drastically (Fig. 1). Nevertheless, the average discharge of the whole August 2022 was lower than in most previous years (Table 1).

**Table 1:** Environmental parameters at the monitoring station of the Brandenburg State Office for the Environment (LfU-BB), Frankfurt/Oder (river km 584; discharge at Eisenhüttenstadt, river km 554) in August, 2017–2022.

Year	T_water_ (°C)	Conductivity (µS cm^−1^)	Turbidity	Nitrate (µg L^−1^)	TP (µg L^−1^)	SRP (µg L^−1^)	TN (µg L^−1^)	Global radiation (W m^−^²)	Discharge (m³ s^−1^)
2017	22.2 ± 1.64	1164 ± 135	24.2 ± 4.0	466 ± 155	179	9	1700	129 ± 36	136 ± 15
2018	24.2 ± 2.47	1240 ± 288	27.9 ± 6.1	325 ± 242	200	7	1100	141 ± 39	83 ± 10
2019	23.4 ± 1.41	1325 ± 244	19.4 ± 12.3	761 ± 184	98	<5	980	159 ± 31	78 ± 7
2020	23.4 ± 1.62	1296 ± 181	15.4 ± 3.4	1889 ± 236	139	31	1700	144 ± 50	154 ± 31
2021	20.8 ± 1.68	1340 ± 198	17.8 ± 5.5	2631 ± 277	128	62	2600	121 ± 43	167 ± 36
2022	23.7 ± 1.22	1623 ± 220	14.8 ± 3.7	990 ± 450	212	27	1320 ± 620	128 ± 41	109 ± 57
p	0.035	0.000	0.0001	0.243				0.241	0.064

Averages and standard deviations of daily means, August 1–31. The significance of the difference between the averages of 2017–2021 and of 2022 is indicated by p-values (t-test). Conductivity, turbidity and nitrate from in situ sensors. Nutrient data (total phosphorus, TP, soluble reactive phosphorus, SRP, total nitrogen, TN) from monthly grab samples. Standard deviation only given for TN in 2022 when sample n = 12.

### Chemical conditions

**Figure 2:**
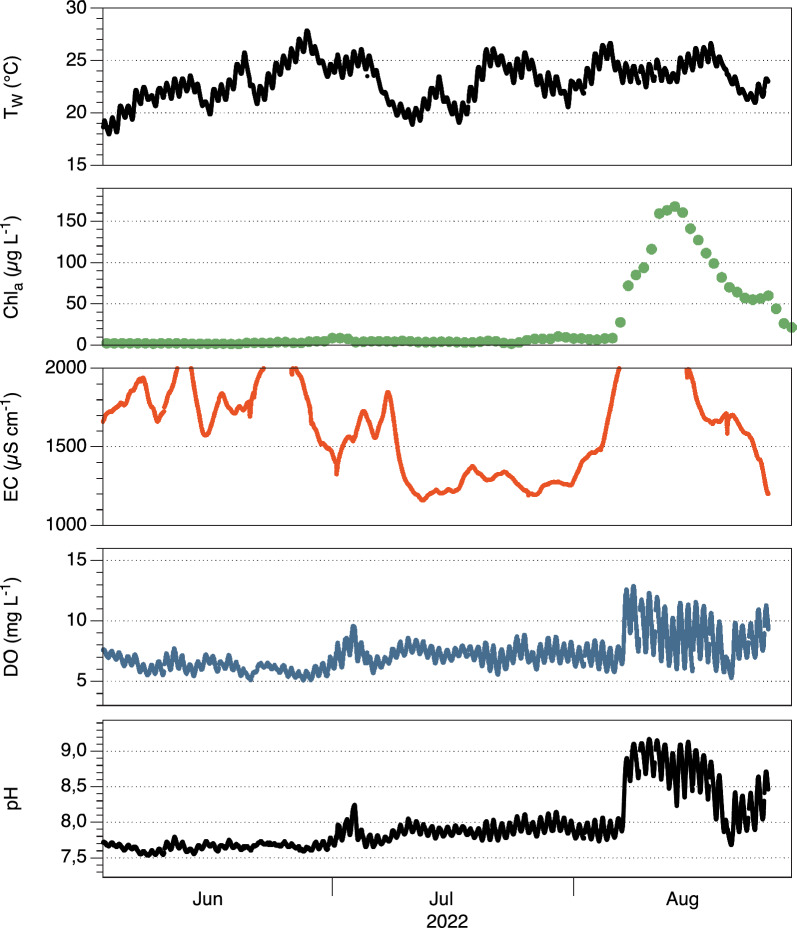
Automated monitoring data in Frankfurt/Oder in July and August 2022. Top to bottom: time series for water temperature (T_w_), chlorophyll-*a* concentration (Chl_*a*_), electrical conductivity (EC), dissolved oxygen (DO), and pH. The EC data is cut off at 2000 µS cm^−1^ due to a range limit of the sensor. Data supplied by the Brandenburg State Office for the Environment (LfU-BB). Chl-*a* data were re-calibrated by HPLC measurements.

Supplementary Fig. S4 provides an overview about sites and dates of water quality measurements. The electrical conductivity (EC) of the river water at Frankfurt/Oder ranged from 401 to 2000 µS cm^−1^ between 2012 and 2022, with an average of 1151 µS cm^−1^ and a weakly positive trend (0.12 µS cm^−1^ day^−1^, R^2^ = 0.15; data supplied by the Brandenburg State Office for the Environment). The significance of this time series is somewhat compromised by the fact that 2000 µS cm^−1^ is the maximum value that the sensor could record^26^. Even with underestimated maxima, the mean conductivity in August was significantly higher in 2022 than in previous years (Table 1). From August 4–24, 2022, EC exceeded 1,500 µS cm^−1^, and from August 6–14, clearly exceeded 2000 µS cm^−1^ (Fig. 2). This latter EC maximum roughly translates to a salinity of 1000 mg L^−1^, which is at the lower end of the brackish water range of 500 to 3000 mg L^−1^, and it can be suspected that peak salinity was even higher. Grab samples taken at Frankfurt/Oder on August 16 confirmed this strongly elevated salinity (sum of major ions 1011 and 1058 mg L^−1^), with sodium chloride being the main solute (708 and 759 mg L^−1^, Supplementary Table S1).

The concentration of nitrate (NO_3_-N) measured at Frankfurt gradually declined from around 5 mg L^−1^ in February to 3 mg L^−1^ in May and to around 1.2 mg L^−1^ in the first week of August. Then, after a slight rise and within a day, the concentration dropped from 1.6 to 0.6 mg L^−1^ on August 7 and remained at this level until August 25 (Supplementary Fig. S5). In the previous 5-year period (2017–2022), the concentration of soluble reactive phosphorus (SRP) ranged from < 5 to 62 µg L^−1^ in grab samples taken in August in Frankfurt. Total phosphorus ranged from 98 to 212 µg L^−1^, and total nitrogen from 980 to 2,600 µg L^−1^ (Table 1). It is noteworthy that while it is difficult to identify a difference in SRP and TP between 2022 and the previous years, nitrate and total nitrogen were depleted, after a year-to-year increase in August concentrations from 2017 to 2021 (Table 1).

### Evidence for phytoplankton mass development

From August 7–26, the Frankfurt/Oder monitoring station recorded characteristic patterns of a massive phytoplankton bloom. The pronounced diurnal oscillations of pH (ca. ± 0.5 pH units, roughly between 8.5 and 9) and dissolved oxygen concentrations (ca. ± 5 mg L^−1^, maximum values at oversaturation > 12.5 mg L^−1^, Fig. 2) reflect the day-night changes in phytoplankton metabolism. Their requirement for nitrogen causes the sharp drop in NO_3_^−^ (Supplementary Fig. S5). Between July 26 and August 6, 2022, the mean Chl-*a* concentration was 8.0 ± 2.5 µg L^−1^. It increased to 72 µg L^−1^ at August 8 and reached a peak from August 12 to 15 (163 ± 4 µg L^−1^). By the end of August, Chl-*a* decreased again to around 45 µg L^−1^ (Fig. 2).

### Propagation of the bloom in the Oder River

**Figure 3:**
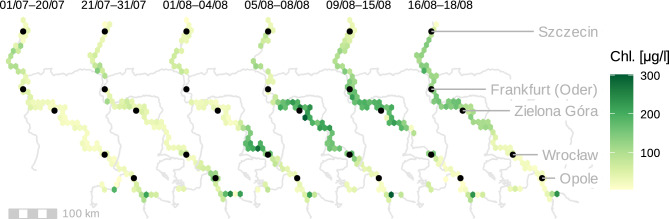
Six satellite-derived Chl-*a* concentration profiles of the Oder River in July and August 2022 depicting the spatial and temporal development of the HAB of *P. parvum*. Note that the time periods for these composite scenes are different in length, because the orbit of Sentinel-2 and occasional cloud cover do not allow for full coverage of the river in a single flyover/a single day. The flow direction is from SE to NW. The four markers at the bottom (upstream Opole) correspond to the Gliwice canal. For an example of the actual resolution of the satellite imagery cf. Supplementary Fig. S10.

**Figure 4:**
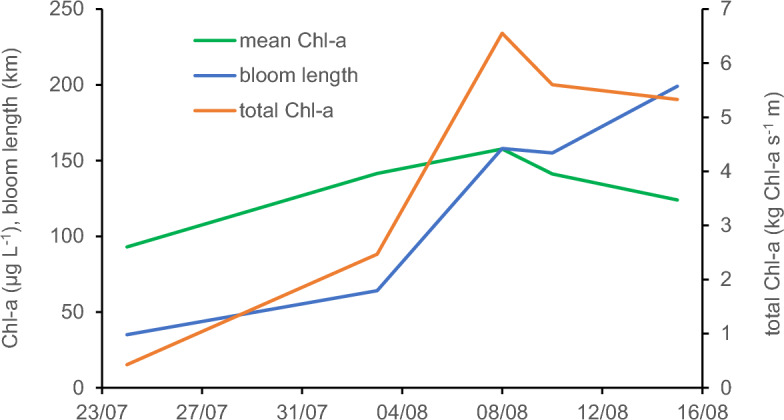
Satellite-derived parameters of phytoplankton development along the Oder River. Total biomass of the bloom (in kg Chl-*a* s^−1^ m), length of the bloom (= distance between sites of 25% and 75% of total biomass in km) and mean Chl-*a* concentration of the bloom.

The spatial distribution of Chl-*a* in the river course over time was reconstructed from optical remote sensing satellite data (Sentinel-2). In the first half of July, elevated Chl-*a* concentrations developed in the Gliwice canal that connects the industrial port of Gliwice to the Oder River at river-km 98. Another phytoplankton hotspot was the Czernica reservoir (river-km 230) with mean Chl-*a* concentrations of 202 µg L^−1^ in the first half of August. In the last week of July, a Chl-*a* peak had developed between river-kms 130 and 190, which progressed towards river-kms 210–360 in the first week of August, and then continued its propagation downstream (Fig. 3 and Supplementary Table 2). During downstream transport, the biomass of the bloom increased about fivefold from July 24–25 to August 3–4 and tripled further until August 8–9 (Fig. 4 and Supplementary Table 2). Due to dispersion and increasing discharge, the mean Chl-*a* concentration of this algal cloud increased much less than the total biomass of the bloom. In mid-August, the HAB had reached its maximum dispersion, and affected the entire lower half of the Oder River, however with already declining mean Chl-*a* (−21%) and total algal biomass (−19%, compared to the maximum at August 8–9, Fig. 4).

### Detection and genetic identification of *Prymnesium parvum*

*Prymnesium parvum* was microscopically detected in samples taken at river km 662 (113,100 cells mL^−1^) on August 15. It was also found in samples taken one day later at km 584 (114,300 cells mL^−1^) and km 614 (116,900 cells mL^−1^). In these three samples, *P. parvum* accounted for 54–57% of the total phytoplankton biovolume. Centric diatoms were second (23–29%), followed by cyanobacteria (10–14% of total phytoplankton biovolume). Three days later, the *P. parvum* abundance at Frankfurt/Oder (river-km 584) was 55,300 cells mL^−1^, and 6 days later already down to 8100 cells mL^−1^ (km 593). On August 27, only about 500 cells mL^−1^ remained (km 593; Fig. 1).

**Figure 5:**
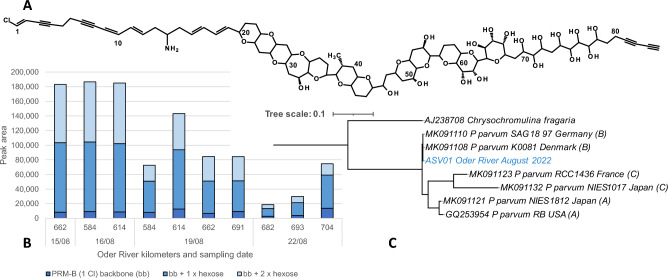
Structure and concentrations of prymnesin and phylogenetic tree. (**A**) B-type prymnesin backbone structure with one incorporated chlorine atom based on^60^. The location and exact identity of the attached one or two hexose moieties was not identified. (**B**) Relative concentration (instrument peak area) of particulate B-type prymnesins obtained from biomass on filter samples. (**C**) ITS-1 (internal transcribed spacer) tree depicting the placement of the Oder-strain of *Prymnesium parvum* in the B-type clade (cf. Supplementary Fig. S6A).

We genotyped *P. parvum* using seven water eDNA samples taken over three days (August 12, 15, 19) from four localities in the Oder River. Sanger sequencing revealed a single nuclear Internal Transcribed Spacer (ITS-1 and -2) genotype (644 bp), that was identical to six sequences on GenBank (Supplementary Fig. S6B) classed as B-type *P. parvum*^27^. DNA metabarcoding of the same samples revealed one dominant ITS-1 genotype (76% of reads) that was identical to the Sanger genotype and other members of a B-type clade (Fig. 5C). Membership of all Oder River samples in the B-type clade was also observed in a gene tree of all available *P. parvum* ITS-1 sequences (Supplementary Fig. S6A). Samples obtained on 12 August from the gills of three haphazardly chosen juvenile sturgeon resulted in the same ITS sequence (644 bp; Supplementary Fig. S7), indicating that *P. parvum* (or at least its DNA) was present in the gills. No A- or C-type genotypes were observed in any samples.

### Identification of *Prymnesium* toxins

The B-type prymnesin backbone with one incorporated chlorine atom and two conjugates thereof, containing one or two additional hexose (but not pentose) moieties, could be detected (Fig. 5A). To our knowledge, this was the first observation that a *Prymnesium parvum* strain produced only three different prymnesin-analoga. The previously investigated strains possessed a more diverse and complex profile^27^. It was shown that the B-type prymnesins were present both in the biomass and whole water samples in physiologically relevant amounts (Fig. 5B, cf.^28^). No A- or C-type prymnesins were detected in any sample. The prymnesin content of particulate matter correlated well with the detected *Prymnesium* biovolume (Supplementary Fig. S8).

### Impact of the event on aquatic communities

The catastrophic fish kill in the Oder River resulted in the death of likely more than 1,000 metric tons of fish, a figure that was extrapolated from a yield of reportedly more than 249 metric tons of dead fish recovered by volunteer efforts along a 160 km stretch of the river in Poland^22^. The majority were larger specimens and species that typically prefer deeper mid-channel sections of the river, including common bream (*Abramis brama*), silver bream (*Blicca bjoerkna*), river gudgeon (*Romanogobio belingii*), perch (*Perca fluviatilis*), and roach (*Rutilus rutilus*). Comparative electrofishing surveys along the riverbanks in the Lower Oder Valley International Park conducted in May and September 2022 at three sites (river-km 667, 687, 703) showed significant density declines of all fish > 10 cm total length independent of species (Supplementary Fig. S9A). A post-event trawl survey in the mid-channel of the lower Oder River in November 2022 confirmed the massive fish loss. Further trawling and electrofishing along the riverbanks revealed stark relative changes of −67% fish density and −64% biomass in the mid channel, and −48% density and −62% biomass at the banks of the middle Oder River (km 544–616). In the lower Oder River (km 618–704), these figures amounted to −53% density and −21% biomass in the mid channel, and + 31% density and −47% biomass at the banks. In addition to fish, mollusks—mussels and gill snails—were also severely impacted; between 47 and 83% of mussels (genera *Anodonta* and *Unio*) and about 90% of snails were killed at some sites. On average, 63% of the mussels died (Supplementary Fig. S9B, C).

## Discussion

**Figure 6:**
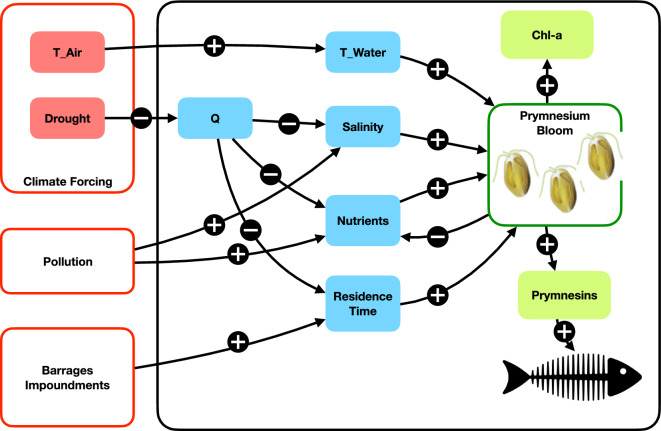
Compounding effects of human impacts and climate forcing (red boxes, left) and consequences for the river ecosystem (black box, right) that lead to the Oder River *Prymnesium* bloom and subsequent fish kill. Symbols on arrows indicate functional positive (+) and negative (−) feedback mechanisms between the entities that they connect. They are supposed to be read in both possible directions—in a sense that positive feedbacks work in the same direction (e.g., air temperature goes up—water temperature goes up; but also: air temperature goes down—water temperature goes down) and negative feedbacks work in the opposite direction (e.g., discharge goes up—salinity goes down because of dilution, and vice versa). Abbreviated parameters: air temperature (T_Air), river runoff (Q), water temperature (T_Water), and chlorophyll-*a* concentration (Chl-*a*).

The massive *Prymnesium* HAB would not have been possible under natural conditions but was enabled by the combination of chronic and acute impacts—chronic high salt load, replete nutrient concentrations, flow velocity reduction through impoundment, and acute climatic forcing including high air and water temperatures and low river discharge (Fig. 6). It extends the established definition of climate-related compound extreme events^29^ by chemical and biological dimensions and is a prime example for compound ecoclimatic events^4^.

Salinity has increased in many river systems during the last decades because of anthropogenic salt input, weathering of construction materials, and geological weathering accelerated by climate change^30^. In the Oder catchment, coal mining in Upper Silesia has entailed many years of intensive discharge of saline mine waters, covered by national legislation. Some tributaries to the Oder River exceed conductivity values of 20 mS cm^–1^ which translates to more than 10‰ salinity^20^ and the river itself typically fluctuates between 1000 and 2000 µS cm^–1^ (https://www.gov.pl/web/odra/badania-odry). The pre-industrial conductivity of the Oder River water is difficult to assess today due to manifold emissions in the catchment, also upstream of the mining region, but one may assume a baseline of 476 ± 69 µS cm^−1^ measured in its upper section at the Czech-Polish border (river km 20, August 21–26, 2022; https://www.gov.pl/web/odra/badania-odry). While the *Prymnesium parvum* complex tolerates a wide range of salinities^31^, the lower growth limit is between 500 and 1000 mg L^−1^^32^ which corresponds to a conductivity of approximately 1000–2000 µS cm^−1^. Mass-developments of *P. parvum* would be therefore impossible under baseline salinity in this river, and even if it would tolerate low salinity, it should have a competitive disadvantage to freshwater algae.

Nutrient excess, in particular of nitrate, has supported the Oder River HAB. The most common natural concentrations in rivers were estimated globally to comprise about 10 µg L^−1^ soluble reactive phosphorus (SRP), 15 µg L^−1^ ammonium, and 100 µg L^−1^ nitrate^33^. Such low nutrient supply would suffice for the assembly of a biomass corresponding to less than 10 µg L^−1^ Chl-*a*. The excessive nitrate concentrations in the Oder River of up to 3 mg L^−1^, while the remaining SRP ranged from 11 to 34 µg L^−1^ during the HAB event, indicates a massive surplus of nitrogen compared to phosphorus. As a mixotrophic species, *Prymnesium* can exploit other algae as a nutrient source, but their biomass would also decline at lower nutrient concentrations.

Very low discharge in this summer (Supplementary Figs. S2 and S3) also favored the algal bloom. Wastewater inflows were less diluted, increasing the concentrations of salt and nutrients compared to mean discharge. It also extended the time of travel available for further phytoplankton growth.

High water temperatures in the Oder River facilitated the proliferation of *P. parvum*. In lakes and reservoirs, *P. parvum* blooms were observed at water temperatures between 10 and 30 °C. In rivers, however, optimal conditions are needed to attain growth rates that exceed the losses caused by dilution. The potentially disastrous HAB “window of opportunity” of phytoplankton is therefore smaller in flushed systems than in standing waters. Namely, *P. parvum* blooms require optimum temperatures (25–30 °C)^31,34^ in rivers. This matches the temperature range that was observed in the Oder River before and during the HAB (Fig. 2 and Table 1).

Strongly regulated river flow and an initial inoculation is another important prerequisite of phytoplankton mass-developments in a river. Assuming a mean flow velocity of 54 km day^−1^ in an unregulated European river^35^ and a mean growth rate of 0.56 day^−1^^32^, a single *P. parvum* cell could produce about 154 daughter cells during travel from the presumed HAB source (ca river-km 100) to Frankfurt/Oder (km 584). An inoculation to the downstream section of almost 5 × 10^16^ *Prymnesium* cells would be needed to result in the observed load of about 7.4 × 10^18^ cells of *P. parvum* during the bloom at Frankfurt. Even if such inoculation contributed only about 1% to the final algal bloom, it is too massive to originate from random sources, such as wind or birds. The water body with long residence time required for inoculation was probably the Gliwice channel that is highly enriched in sodium chloride from mining emissions^22^. Actually, high Chl-*a* concentrations had been observed in this channel in July–August 2022 (Fig. 3). At the same time, low-flow conditions in the river downstream of the inoculation enabled ongoing growth of *P. parvum*, as suggested by indicators of diurnal metabolism (pH, dissolved oxygen, Fig. 2). Accordingly, extracted river Chl-*a* profiles support a "wave-like" initial transport, dispersal and proliferation, and breakdown of the HAB (Figs. 1 and 4, Supplementary Table S2).

### Short-term perspectives for the ecosystem

If extensive discharge of salts and nutrients are not stopped and reservoirs and impoundments are not removed, future *Prymnesium* blooms will only require suitable temperatures and a prolonged low-flow situation. The dramatic losses of large filter feeders in 2022 make the system more susceptible to future algal blooms. Remaining vegetative cells or resting stages from the 2022 *Prymnesium* bloom may strongly increase the likelihood of a new HAB. Any additional HAB would interrupt the recovery of the populations of fish and benthic filter-feeders damaged in 2022. However, with immediate and significant reductions in salt loads and accelerated efforts to reduce nutrient loads, the fish populations could recover within three to four years^36,37^. Mussels will probably recover much more slowly, taking more than ten years for the system to return to its pre-catastrophic ecological state^7^. *Prymnesium* toxins affect zooplankton in species-specific ways, potentially causing shifts in the food web^38^. Prolonged periods of low discharge and elevated water temperatures as witnessed in 2022 will come again and favor plankton growth via extended residence time and improved light supply. These summer conditions, which were a 1 in 100 event in pre-industrial times, are already expected today to occur on average every 20 years and these return times will shorten substantially with progressing climate change^25^.

### Lessons from the 2022 Oder River environmental disaster

The catastrophic fish and mollusk kill of the Oder River HAB was the result of combined human impacts that we perceive as a blueprint for unforeseen but rising threats to many large river ecosystems worldwide. Our current evidence highlights the vulnerability of pre-stressed ecosystems to changing environmental forcing and emphasizes the need for immediate action to relieve anthropogenic pressure—in this case, reduce salt and nutrient loads, and prevent further channelization and dam construction. Information on the genetic prerequisites and ecological preferences of the Oder-*Prymnesium* strain will help assessing the recovery potential of the Oder River system and are currently being obtained in the framework of a large project (https://oder-so.info/en). Like the Oder River, almost all aquatic ecosystems globally are already subject to anthropogenic stresses, including salinization, engineering (dredging, dam construction), wastewater emission, and invasive species. But these preconditions will be exacerbated under climate change and have the potential to push ecosystems towards collapse^39^. Climate-driven multivariate compound effects are occurring with increasing frequency and have devastating consequences for aquatic ecosystems^5^. While they have been poorly considered in impact models of climate change to date, we expect them to become the new “normal” if precautions are neglected.

## Materials and methods

### Study site

The Oder River is an 866 km long lowland river with a catchment of 118,890 km^2^ in the Czech Republic, Poland, and Germany. While the upper Oder River is impounded over 204 km length and fragmented by 25 weirs, its middle and lower course from river-km 300 is free flowing without any barrier to the Baltic Sea and includes several regions of extraordinary importance for ecological conservation. The Oder River is highly eutrophic and has been affected by salt emissions from coal and ore mining in the upper catchment (Upper Silesia^20^). The European Pollutant Release and Transfer Register lists 34 facilities with high chloride discharge, especially around the Gliwice canal^21^.

### Sample collection, field measurements, and public data

Nine grab water samples were collected with rope and bucket from the free-flowing center of the Oder River, using bridges at seven sites between August 16 to 22, 2022, at and shortly after the algal peak (Supplementary Fig. S4). One additional water sample was obtained from a sturgeon rearing facility at Friedrichsthal (August 12) and one was provided by the Brandenburg State Office for the Environment (Frankfurt, August 15). In the bucket samples, water temperature (T), pH value, electric conductivity (EC) and dissolved oxygen concentration and saturation were determined with a hand-held multiprobe (WTW multi 3630i). Subsamples for general water chemistry were filled in 1 L HDPE bottles, and subsamples for *Prymnesium* identification and prymnesin analysis were filled in 1 L borosilicate glass bottles. These samples were brought to the lab within 2 h after retrieval and processed, conserved, and stored for further analysis. Additionally, records of the automatic monitoring station at Frankfurt/Oder were analyzed. This station is run by the Brandenburg State Office for the Environment and measured conductivity, air and water temperature, global radiation, Chl-*a*, pH, nitrate and oxygen concentrations every ten minutes (https://lfu.brandenburg.de/lfu/de/aufgaben/wasser/fliessgewaesser-und-seen/gewaesserueberwachung/wasserguetemessnetz/frankfurt-oder/). These data are complemented by monthly grab samples that are analyzed in the state laboratory for various parameters including total phosphorus (TP), soluble reactive phosphorus (SRP) and total nitrogen (TN).

### Water quality analyses

Dissolved nutrients ammonium (NH_4_-N), nitrate (NO_3_-N), and soluble reactive phosphorus (SRP) were determined by flow segmented analysis (SEAL AA3) in membrane filtered (0.45 µm) and acidified (2 M HCl) samples. Total phosphorus (TP) was determined in original samples by molybdenum blue colorimetry after wet-chemical digestion with hydrogen peroxide and sulfuric acid. Total carbon (TC) and total nitrogen (TN) were determined in unfiltered and acidified (2 M HCl) samples by catalytic oxidation and infrared spectroscopy and chemiluminescence. Sulfate (SO_4_^2−^) and chloride (Cl^–^) were determined by ion chromatography, conductivity detection after chemical suppression (Metrohm CompactIC) in membrane filtered (0.2 µm) samples. Sodium (Na), potassium (K), calcium (Ca) and magnesium (Mg) were determined in membrane filtered (0.45 µm) and acidified (2 M HCl) samples by inductively-coupled plasma optical emission spectroscopy.

### Analyses of the phytoplankton dynamics

Phytoplankton species composition was analysed in living and in Lugol fixed subsamples. Species abundance was enumerated by inverted microscopy following^40^. Species-specific cell volumes were estimated from measurements of all dimensions from at least 20 cells per species, assuming simple geometric shapes. Other subsamples were filtered onto GF/F glass-fibre filters which were immediately frozen at −80 °C. Pigment analyses were performed by HPLC after freeze-drying and extraction with dimethylformamide (DMF). For details of the chlorophyll-*a* analyses see^41^.

### DNA extraction and genotypic characterization

*Prymnesium* were genotyped from water samples using Sanger sequencing of the nuclear ITS region, and DNA metabarcoding with next-generation sequencing of the ITS-1 region as follows. Water samples were taken on August 15 and 19, 2022 for a total of seven samples from four sites (river-kms 584, 614, 662, and 691). DNA was extracted from filters using a DNeasyPowerSoil Pro kit (Qiagen, Hilden, Germany). We also genotyped *Prymnesium* from three tissue samples taken from dead juvenile sturgeon (*Acipenser oxyrinchus*) that had died after direct exposure to river water on August 12, 2022 in a restocking tank of a rearing facility situated next to the river near Friedrichsthal (river-km 706). These individuals belonged to the German conservation and species restoration program of the Oder River^42^. We haphazardly selected three individual sturgeons of ca. 4 cm body length, removed ca. 0.25 g of gill tissue, extracted DNA using the DNeasy Blood and Tissue Kit (Qiagen), and Sanger-sequenced the ITS region.

For Sanger sequencing, PCR (95 °C for 2 min; 38 cycles of 95 °C for 2 min, 69 °C for 1 min, 72 °C for 1 min; 72 °C for 5 min) reactions contained 5 μL 5× AllTaq buffer (Qiagen), 0.5 μL dNTPs (Agilent), 1.25 μL of each primer, 0.5 μL of AllTaq (Qiagen), and 2 μL DNA (concentration reaching from 10 to 88 ng μL^−1^), and 14.5 μL RO-filtered water. We newly designed *Prymnesium*-specific primers (Prym_ITS_F: 5'_CCGGTCTTTCCACCCACCA_3'; Prym_ITS_R: 5'_GCCCACCGGTACGCCTCG_3') using published sequences (GenBank accessions MK091108-MK091133^27^. This approach was also applied to minced gills samples of dead juvenile sturgeon (see above) to avoid potential interaction with the sturgeon genomic DNA. In this case, we also tested dilutions of 1:10, and 1:100 as templates. Products were purified and sequenced in both directions using the same primers at Eurofins Genomics, manually edited, and aligned with the *Prymnesium* sequences from^27^. ITS gene trees for simple sequence assignment were reconstructed using PhyML^43^ with neighbor-joining and 100 bootstrap iterations. Sequence data are available at the GenBank Accession numbers PP426008–PP426014.

Next-generation sequencing was carried out on an i7 Hybrid liquid-handling robot (Beckmann-Coulter) at the Berlin center for Genomics in Biodiversity Research. PCR reactions (98 °C for 60 s; 30 cycles of 98 °C for 10 s, 67 °C for 20 s, 72 °C for 10 s; 72 °C for 2 min) included 10 ng template DNA, 12 μL Q5 High-Fidelity Master Mix (NEB), 1.25 μL each of primers that were designed for the ITS-1 region using PriSeT^44^, and RO-filtered water for a total reaction volume of 25 μL. PCR products were cleaned using a magnetic bead protocol (Agencourt AMPure XP, Beckman Coulter, Indianapolis, IN, USA). DNA concentration was measured using the QuantiFluor^®^ dsDNA System (Promega, Madison, WI, USA), and PCR products all were normalized to a concentration of 5 ng μL^−1^. An indexing PCR reaction (95 °C for 2 min; 8 cycles of 95 °C for 20 s, 52 °C for 30 s, 72 °C for 30 s; 72 °C for 3 min) was used to add unique 12-bp inline sequence barcodes (Nextera Index Kit, Illumina, San Diego, CA, USA) to each sample, using 10 μL of target PCR product and with 5 μL reaction buffer (Herculase II Fusion DNA Polymerase, Agilent), 0.25 μL dNTPs (Agilent), 0.625 μL each of index primers P5 and P7 (Nextera Index Kit, Illumina), 0.25 μL polymerase (Herculase II Fusion DNA Polymerase, Agilent), 1 μL DMSO, and RO-filtered water for a total reaction volume of 25 μL. PCR products were purified and quantified as above. Samples were then pooled in equimolar amounts and sequenced on an Illumina MiSeq using a v3 sequencing kit (600 cycles). Negative controls were included as part of all PCR reactions and were sequenced in the same run as the regular samples. Raw sequence data (fastq.gz files) are available at the Sequence Read Archive BioProject accession number ID: PRJNA1090943.

Raw sequence data were analyzed using the DADA2 v 1.28.0 package^45^ in R (Team R, 2023). The complete R script is provided as a supplementary file (.R). Taxonomic assignment of amplicon sequence variants (ASVs) was performed using the assignTaxonomy function with default parameters in DADA2, and the Protist Ribosomal Reference (PR2 version 4.12.0) database^46^.

### Prymnesin analysis

Extraction of the biomass on filter samples or the water samples collected at different locations and times along the German side of the Oder River (see Supplementary Figure S4) was performed as described in^28^ with small modifications as described in^47^. In brief, the filters containing the biomass were transferred to 15 mL polypropylene tubes and were extracted twice with 12 mL HPLC-grade methanol in the ultrasonic bath for 30 min. The samples were centrifuged at 3220 *g* for 10 min and the supernatants were combined and dried using a CentriVap Benchtop Vacuum Concentrator coupled to a CentriVapColdtrap (both Labconco Corporation, Kansas City, MO, USA) at 10 °C. For reconstitution 0.5 mL methanol (MeOH):H_2_O (90:10, v/v) was used. The water samples with or without biomass (90 mL each) were extracted by liquid–liquid extraction using pure 2-butanol in the following way: First, 3.42 g of NaCl were added to the water samples with or without biomass to increase the extraction efficiency of prymnesins. Thereafter, in the first extraction step 40 mL solvent and in the two consecutive extractions 20 mL each were used. The resulting three organic phases were combined, washed twice with 20 mL H_2_O and dried down as described above for the biomass samples. Finally, the samples were reconstituted in 0.5 mL MeOH:H_2_O (90:10, v/v). The presence and identity of the prymnesins were performed by liquid chromatography-mass spectrometric measurements using a Vanquish HPLC-system (Thermo Scientific, Waltham, MA, USA) coupled to a timsTOF flex system (Bruker Corporation, Billerica, MA, USA). The mass spectrometer was operated in positive electrospray ionization mode and data were collected from *m/z* 100 to 2000. A Kinetex F5 (2.1 × 100 mm, 2.6 µm, Phenomenex, Aschaffenburg, Germany) column equipped with a guard column of the same type was used together with a water-acetonitrile gradient both containing 0.1% formic acid and 1 mM ammonium formate. The instrument was controlled with Qtof Control Version 6.2 (Build 4.0), Compass HyStar 6.0 (Version 6.0.30.0) (both Bruker) and Chromeleon 7.3 (Thermo Scientific). The estimation of the prymnesin concentrations were performed with the indirect method described in^28^ using the AccQ-Fluor reagent WAT052880 (Waters Corporation, Milford, MA USA) and the mycotoxins fumonisin B_1_ and B_2_ (RomerLabs, Tulln, Austria) for external calibration. A 1200 series HPLC system (Agilent Technologies, Waldbronn, Germany) was used together with an Agilent Poroshell 120 EC-C18 column (2.1 × 50 mm, 2.7 µm) and a water-acetonitrile gradient (both with 0.1% formic acid). Data evaluation was performed with Bruker Compass DataAnalysis Version 5.3. and Excel^®^Microsoft Corporation. All previously detected and proposed prymnesins according to^27^ were investigated, but only three B-type prymnesins could be detected in the Oder River samples (Supplementary Table S3). The backbone of the identified B-type prymnesin is provided in Fig. 5A, the exact location of the hexose conjugates could not be determined with the applied method.

### Analysis of Sentinel-2 MSI data

Chl-a concentration were derived from Sentinel-2's multispectral instrument (MSI) satellite data available for the time span July and August 2022, using the European Space Agencies (ESA) Sentinel Application Platform (SNAP) toolboxes. The Sentinel-2 L1C products were resampled to 20 m spatial resolution for all spectral bands by applying the S2Rsampling processor. Water pixels were separated from land, cloud, and cloud shadow pixels by using the Idepix processor^48^. Atmospheric correction and in-water retrieval were performed by applying the C2RCC processor which relies on a large database^49,50^. The processor offers neural networks trained for different water types. Version C2X-COMPLEX was applied.

### Extraction of river chlorophyll concentration profiles

To calculate the Chl-*a* profiles along the length of the river network, data derived from Sentinel-2 (Supplementary Fig. S10) were matched to the river network (INSPIRE^51^). The raw data were transformed into a connected graph and a spatial network to match Chl-*a* and geographic position. All calculations were done in R (v4.3.1)^52^, using the packages sf (v1.0-14)^53^, sfnetworks (v0.6.3)^54^, and igraph (v1.5.1)^55^ for the network analysis. River discharge data was obtained from the European Flood Awareness System (EFAS), the EFAS Historical Reanalysis Data version 4.0 was used to estimate river discharge^56^. Measured river discharge data was obtained from Brandenburg State Office for the Environment and from^19^. The scripts of this data analysis are available in a permanent repository under 10.5281/zenodo.8343700. Chl-*a* concentration, dispersion of the algal bloom and discharge changed along the river at the same time (see Supplementary Fig. 11). To estimate the total biomass of the algal bloom, the Chl-*a* load (= concentration x discharge) was calculated per section between adjacent Chl-*a* data points, multiplied by the length of the section and integrated over all sections with Chl-*a* higher than background concentrations.

### Mussels and fish sampling

Mussels were sampled in three groyne fields in the lower River Oder National park. Within each groyne field ten 1 m^2^ plots had been sieved until no additional mussel was found in June 2022. All mussels were determined to taxon, counted and their densities estimated. After the Oder River catastrophe, the next groyne fields upstream of those sampled in June had been similarly sampled on August 24, 2022. In addition to previous metrics, living and freshly killed mussels had been counted separately. Sampling details have been reported in^57^.

Fish were sampled by trawling in the mid-channel section and by electric fishing along the banks^58,59^. Trawling was performed using a bottom otter trawl pulled downstream at 8 km h^−1^ speed over ground for one or two kilometers^58^. Boat electric fishing was performed single pass, in upstream direction using a generator-powered DC electric fishing gear (Type FEG 8000) with handheld ring anode. Usual length fished were at least 400 m or the complete bank length if a particular bank habitat type was shorter^58^. All captured fish were identified, measured for total length and weighted or body mass was back-calculated from length. Lower and middle Oder River have been comparably sampled for fish since 1998 and 2006, respectively. In total 1103 trawl and 264 electric fishing samples were taken between river-kms 544–616 and 618–704 before the fish kills in August 2022 and 93 trawl and 52 electric fishing samples thereafter. These data (details in^59^) enabled assessing fish losses against their natural population variation and dynamics.

### Supplementary Information

Supplementary Information.

## Data Availability

Supporting figures and tables are displayed in a Supplementary Information file. The data analyzed and presented during this study are made available through the freshwater and environmental research database (FRED, https://fred.igb-berlin.de/data/package/890).
