# Supplementary material for: Unpredicted ecosystem response to compound human impacts in a European river
Source: Sci Rep. 2024 Jul 16;14:16445. doi: 10.1038/s41598-024-66943-9 (PMC11252402; doi:10.1038/s41598-024-66943-9)
Supplement: Supplementary file 1 — Supplementary Information. [file 41598_2024_66943_MOESM1_ESM.docx]

Supplementary Materials for

**Unpredicted ecosystem response to compound human impacts in a European river**

Jan Köhler, Elisabeth Varga, Stephanie Spahr, Jörn Gessner, Kerstin Stelzer, Gunnar Brandt, Miguel D. Mahecha, Guido Kraemer, Martin Pusch, Christian Wolter, Michael T. Monaghan, Matthias Stöck, Tobias Goldhammer

Corresponding authors' Email: jan.koehler@igb-berlin.de, elisabeth.varga@vetmeduni.ac.at, tobias.goldhammer@igb-berlin.de

**This PDF file includes:**

Supplementary Figures S1 to S11

Supplementary Tables S1 to S3

Fig. S1: Map of the Oder River and its major tributaries, important cities, and sampling locations.

Fig. S2. Cumulative discharge between June 1 and August 15 by year for 7 different locations in the Oder River basin. Data for Eisenhüttenstadt and Hohensaaten-Finow are measured directly (1963-2022, data source: Brandenburg State Office for the Environment, 2022), the other 5 locations are re-analysis data (1991-2022, data source: EFAS 4.0 historical re-analysis data). The red lines indicate data of 2022.

**Fig. S3.** Daily means of discharge at river km 554 (Eisenhüttenstadt) from July till mid-September. Solid line: 2022, dotted line: mean discharge from 2000-2021, dashed line: minimum discharge from 2000-2021.

**Fig. S4:** Dates and sites of sampling along the lower Oder River during the *Prymnesium* bloom (indicated by the square), August-September 2022. Red lines = automatic monitoring stations. Full = analyses of algal abundance, Chl-a, nutrients, conductivity, and toxin content as well as sequencing.


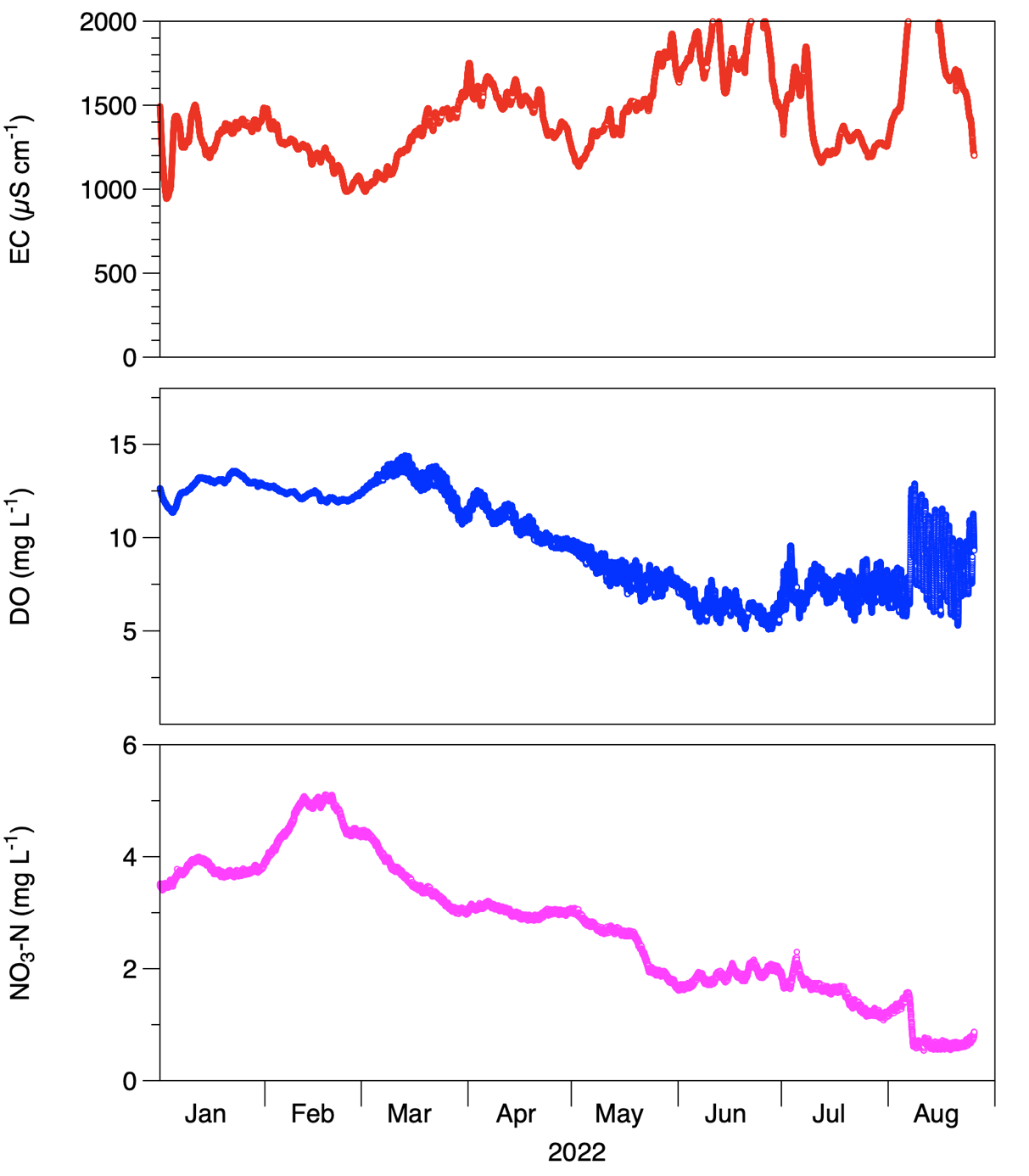


Fig. S5. Extended time series (January to August 2022) for electric conductivity (EC), dissolved oxygen (DO) and nitrate-nitrogen (NO_3_-N) measured at the automated monitoring station Frankfurt/Oder. The EC data is cut off at 2000 µS cm^-1^ due to a range limit of the sensor. Data supplied by the Brandenburg State Office for the Environment (LfU-BB).


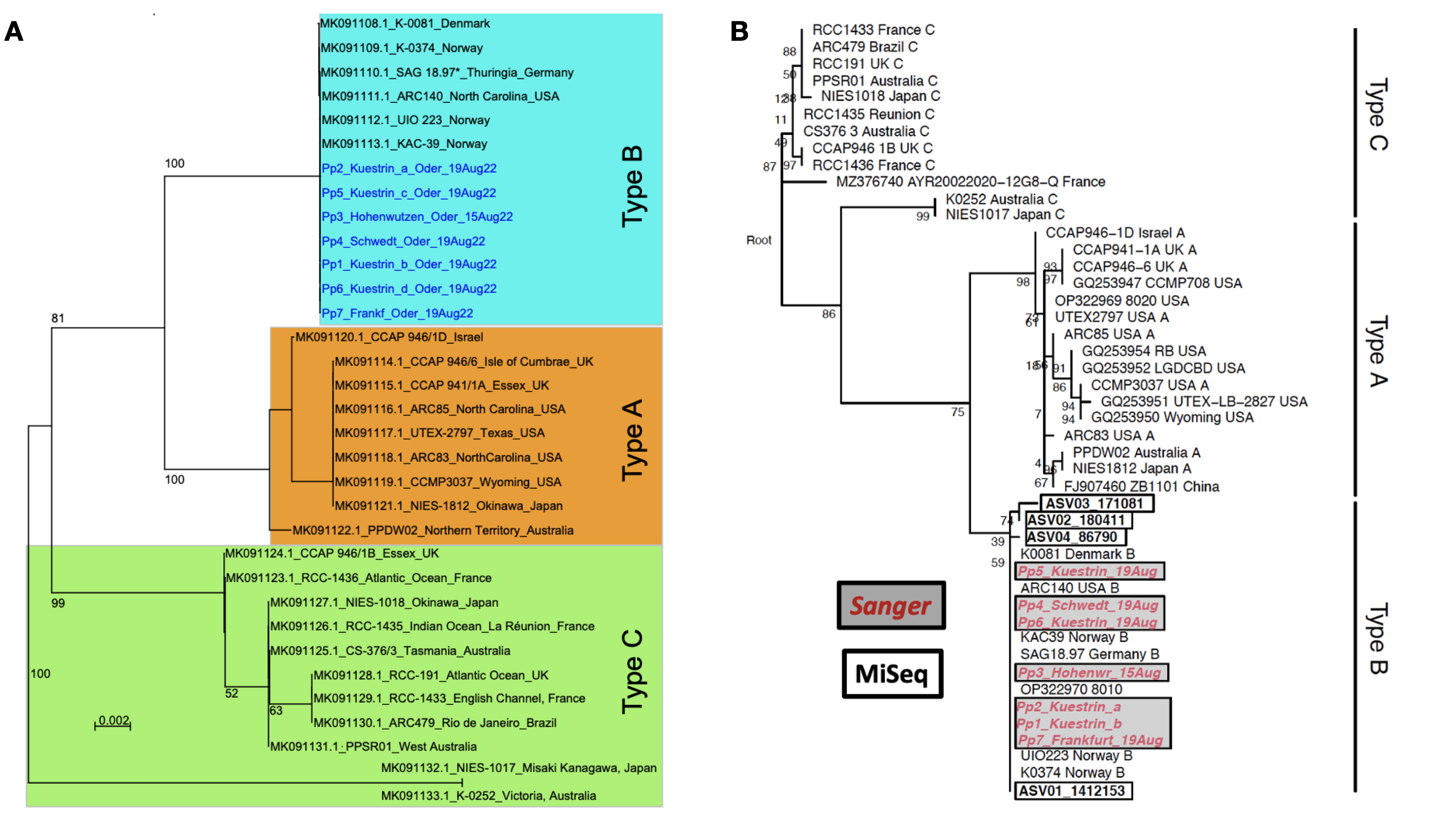


Fig. S6. (A) Initial tree obtained from ITS1- and ITS2-sequences (644 bp) amplified using new primers Prym_ITS_F and Prym_ITS_R from the Oder River *Prymnesium*, including water and gill samples of dead juvenile sturgeon (see Methods) from September 10, 2022, obtained by Sanger-sequencing. (B) Phylogenetic assignment of the Oder River genotype of *P. parvum* using globally distributed strains with internal transcribed spacer (ITS-1) sequences available on GenBank. Each leaf shows the GenBank accession number, followed by the strain abbreviation, geographic region and country. Numbers at main branches show percentages of bootstrap support from 100 resampled datasets. Results from Sanger (grey boxes with red letters) and next-generation sequencing (white boxes).


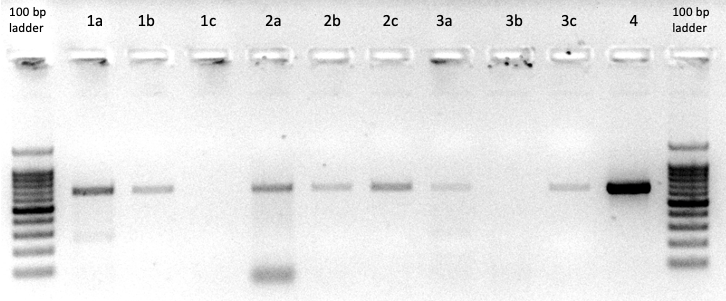
Fig. S7. Results of a PCR using the newly designed primers Prym_ITS_F and _R in three randomly chosen dead juvenile sturgeon (*Acipenser oxyrinchus*). These fish died in the tanks (which are connected to the Oder River) at Friedrichsthal on August 12, 2022.

1a-c, 2a-c, and 3a-c show PCR-amplicons from gill-DNA extractions with a) pure and b) 1:10 and c 1:100 dilutions; 4 shows a positive control of *Prymnesium parvum* DNA. This 1.5% agarose gel image was stained using ethidium bromide (7.5 μL/250 mL) and visualized with the software Gelimage; no further image manipulation was used.

Fig. S8. Correlation of prymnesin extracted from particulate material (relative concentrations, see Methods) against *Prymnesium* biovolume (BV). Phytoplankton was counted by inverted microscopy in Lugol fixed subsamples. Species-specific cell volumes were estimated from measurements of all dimensions from at least 20 cells per species, assuming simple geometric shapes.


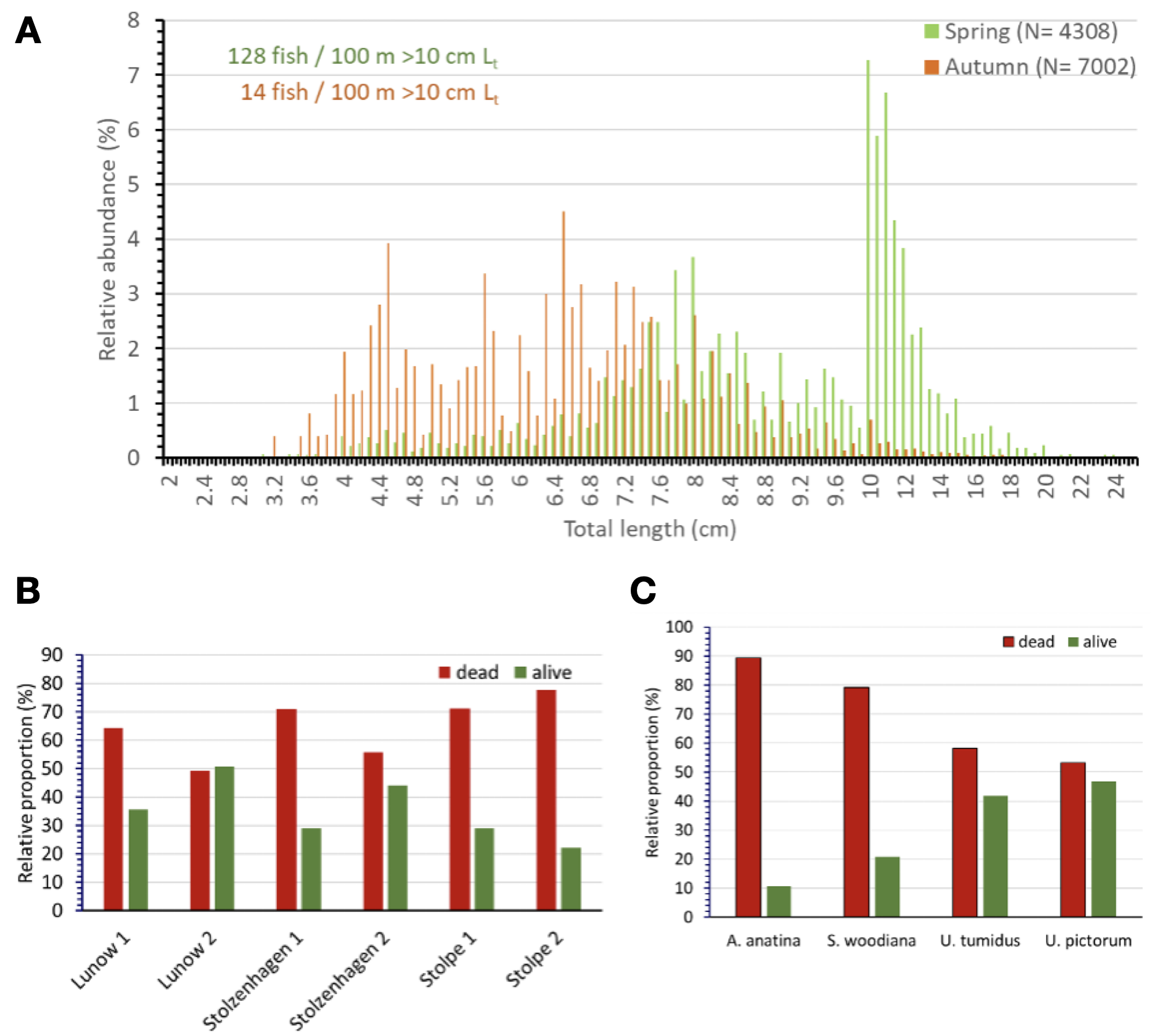


Fig. S9. (A) Fish size distribution and relative abundance of bigger fish (>10 cm total length) at three sites in the International Park “Lower Oder Valley” in September 2022 compared to May 2022. (B) Proportions of dead and alive mussels (N= 708) on
24 August 2022 at six 1 m² sample sites in three groyne fields (C) Proportions of dead and alive mussels according to genera: *A.= Anodonta, S.= Sinanodonta, U.*= *Unio*.


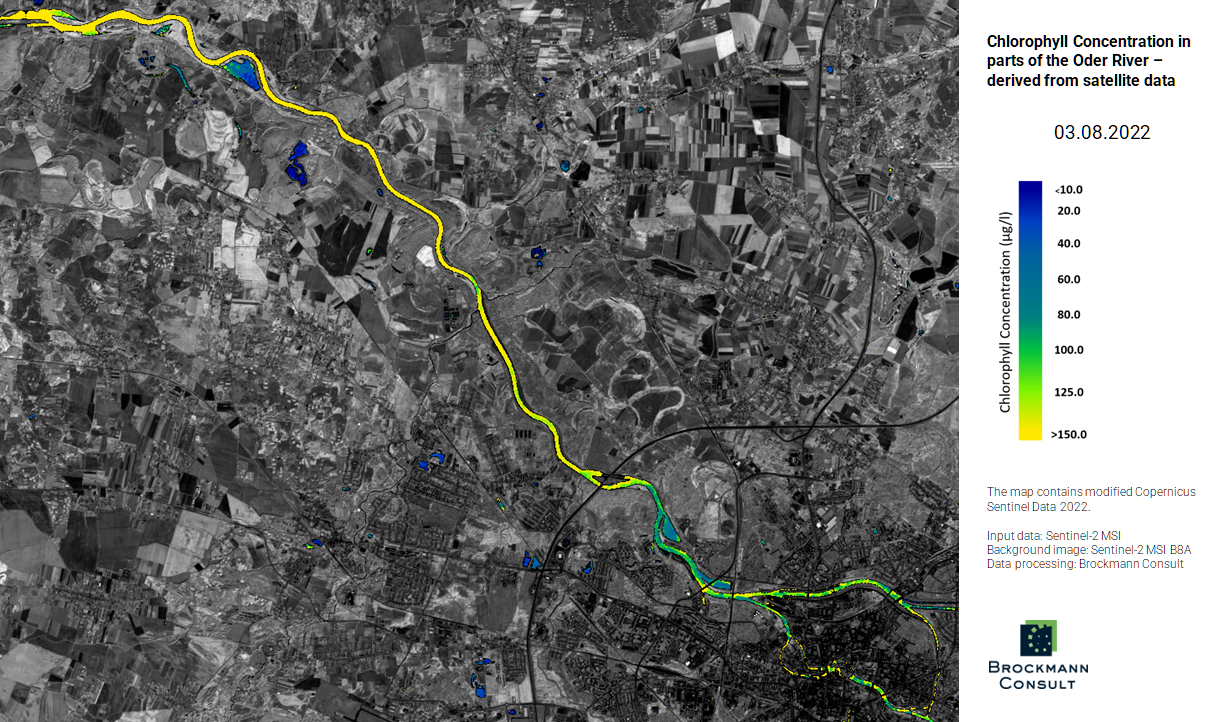

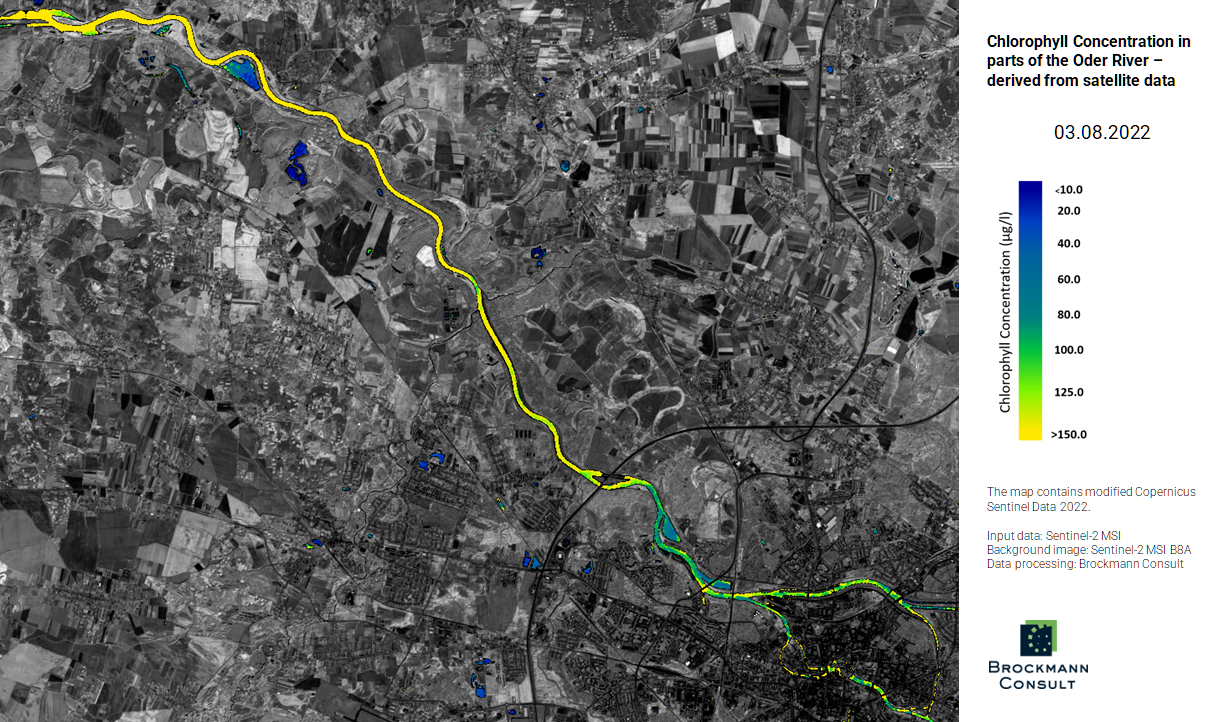


Fig. S10. Example of chlorophyll concentration derived from Sentinel–2 data of the Oder River downstream of Wrocław (bottom right corner) for the satellite acquisition from August 3, 2022. Water bodies including the Oder River are visualized in color according to the chlorophyll color scale, the surrounding landscape is in grey scale.

Fig. S11. Chlorophyll concentration (green) extracted from satellite data and discharge anomalies (blue) derived from EFAS historical reanalysis data by distance from the mouth of the Oder river. The four consecutive chlorophyll concentration profiles in August (middle and bottom panels) suggest build-up, propagation, dispersal and breakdown of a “chlorophyll wave” during transport in the river in relation to episodic changes in discharge.

Table S1. Water quality components measured in grab water samples taken on various locations in the Oder during the event in August 2022. The parameters were measured as follows: pH, temperature (T), electric conductivity (EC) with a hand-held multiprobe (WTW multi 3630i, Xylem Analytics, Germany) in the field; total carbon (TC) and total nitrogen (TN) by catalytic oxidation infrared spectroscopy and chemiluminescence (TN only); total phosphorus (TP) by wet chemical oxidation and molybdenum blue photometry; ammonium-nitrogen (NH_4_-N), nitrate-nitrogen (NO_3_-N), and soluble reactive phosphorus (SRP) by colorimetric flow-segmented analysis; chloride (Cl^–^) and sulfate (SO_4_^2-^) by ion chromatography; calcium (Ca), potassium (K), magnesium (Mg) and sodium (Na) by inductively coupled plasma-optical emission spectroscopy (see Methods).

| Sampling Site | Date | River km | Coordinates | pH | T | EC | TC | TN | TP | SRP | NH_4_–N | NO_3_–N | Cl^–^ | SO_4_^2-^ | Ca | K | Mg | Na |
| --- | --- | --- | --- | --- | --- | --- | --- | --- | --- | --- | --- | --- | --- | --- | --- | --- | --- | --- |
|  |  |  |  |  | *°C* | *µS cm^-1^* | *mg L^-1^* | | *µg L^-1^* | | *mg L^-1^* | | | | | | | |
| Frankfurt | 08/16 | 584 | 52.348269N 14.556207E | 9.24 | 28.1 | 1854 | 10.4 | 0.8 | 266 | 11 | 0.04 | 0.16 | 454 | 117 | 74 | 11.1 | 19.3 | 253.5 |
| Küstrin | 08/16 | 614 | 52.579084N 14.628883E | 9.44 | 26.8 | 1987 | 9.9 | 0.8 | 276 | 18 | 0.02 | 0.02 | 490 | 119 | 75 | 10.8 | 19.0 | 268.2 |
| Frankfurt | 08/19 | 584 | 52.348269N 14.556207E | 8.79 | 25.2 | 1650 | 6.4 | 0.6 | 266 | 31 | 0.04 | <0.01 | 374 | 118 | 77 | 10.4 | 19.3 | 198.3 |
| Küstrin | 08/19 | 614 | 52.579084N 14.628883E | 8.97 | 25.5 | 1679 | 7.0 | 0.7 | 258 | 37 | 0.04 | <0.01 | 385 | 117 | 76 | 10.1 | 19.2 | 201.4 |
| Hohenwutzen | 08/19 | 662 | 52.843231N 14.123260E | 9.37 | 25.2 | 1160 | 6.9 | 0.6 | 299 | 28 | 0.04 | <0.01 | 226 | 82 | 69 | 7.8 | 13.9 | 128.4 |
| Schwedt | 08/19 | 691 | 53.034468N 14.312136E | 8.67 | 26.1 | 1207 | 6.9 | 0.8 | 239 | 34 | 0.04 | 0.01 | 245 | 81 | 70 | 8.1 | 13.9 | 147.6 |
| Piasek | 08/22 | 682 | 52.988263N 14.215633E | 8.6 | 22.3 | 1145 | 6.2 | 0.6 | 253 | 29 | 0.03 | 0.02 | 224 | 82 | 72 | 7.7 | 14.4 | 121.7 |
| Schwedt | 08/22 | 693 | 53.048880N 14.340312E | 8.6 | 22.6 | 1137 | 7.0 | 0.6 | 196 | 23 | 0.03 | 0.01 | 199 | 74 | 67 | 7.2 | 13.3 | 114.4 |
| Branch Westoder | 08/22 | 704 | 53.141290N 14.386915E | 8.6 | 23.2 | 1148 | 7.0 | 0.8 | 214 | 20 | 0.03 | 0.01 | 219 | 85 | 71 | 7.9 | 14.2 | 126.0 |

**Table S2**: **Satellite-derived indices of phytoplankton development along the Oder river.** Relative biomass of the bloom (area of the Chl-a peak x discharge, maximum bloom biomass at August 8-9 = 100%, and position of the bloom at river kilometers
(cf. Supplementary Figure S11) with 25-, 50- and 75-percentiles of the bloom passed.

| Date | Relative bloom biomass in % | 25% of bloom at km | 50% of bloom at km | 75% of bloom at km |
| --- | --- | --- | --- | --- |
| July 24-25 | 7 | 143 | 159 | 178 |
| Aug 3-4 | 38 | 254 | 282 | 318 |
| Aug 8-9 | 100 | 332 | 415 | 490 |
| Aug 10-11 | 86 | 392 | 473 | 547 |
| Aug 14-16 | 81 | 408 | 504 | 607 |

Table S3. Overview over the detected B-type prymnesins including their proposed formulae and exact masses.

| Systematic abbreviation | proposed formula | Mass (neutral) | [M+H]^+^ | [M+2H]^+2^ | [M+Na+H]^+2^ | [M+H+NH_4_]^+2^ |
| --- | --- | --- | --- | --- | --- | --- |
| PRM-B (1 Cl) | C_85_H_122_ClNO_29_ | 1655.7791 | 1656.7864 | 828.8968 | 839.8878 | 837.4101 |
| PRM-B (1 Cl) + hexose | C_91_H_132_ClNO_34_ | 1817.8319 | 1818.8392 | 909.9232 | 920.9142 | 918.4365 |
| PRM-B (1 Cl) + 2 hexose | C_97_H_142_ClNO_39_ | 1979.8848 | 1980.8920 | 990.9497 | 1001.9406 | 999.4629 |
